# Microbial interactions play an important role in regulating the effects of plant species on soil bacterial diversity

**DOI:** 10.3389/fmicb.2022.984200

**Published:** 2022-09-15

**Authors:** Yajun Wang, Lan Ma, Ziyang Liu, Jingwei Chen, Hongxian Song, Jiajia Wang, Hanwen Cui, Zi Yang, Sa Xiao, Kun Liu, Lizhe An, Shuyan Chen

**Affiliations:** ^1^School of Life Sciences, Lanzhou University, Lanzhou, Gansu, China; ^2^College of Ecology, Lanzhou University, Lanzhou, Gansu, China

**Keywords:** bacterial diversity, structural equation model, antagonism, microbial interactions, plant species

## Abstract

Plant species and microbial interactions have significant impacts on the diversity of bacterial communities. However, few studies have explored interactions among these factors, such the role of microbial interactions in regulating the effects of plant species on soil bacterial diversity. We assumed that plant species not only affect bacterial community diversity directly, but also influence bacterial community diversity indirectly through changing microbial interactions. Specifically, we collected soil samples associated with three different plant species, one evergreen shrub (*Rhododendron simsii*) and the other two deciduous shrubs (*Dasiphora fruticosa* and *Salix oritrepha*). Soil bacterial community composition and diversity were examined by high-throughput sequencing. Moreover, soil bacterial antagonistic interactions and soil edaphic characteristics were evaluated. We used structural equation modeling (SEM) to disentangle and compare the direct effect of different plant species on soil bacterial community diversity, and their indirect effects through influence on soil edaphic characteristics and microbial antagonistic interactions. The results showed that (1) Plant species effects on soil bacterial diversity were significant; (2) Plant species effects on soil microbial antagonistic interactions were significant; and (3) there was not only a significant direct plant species effect on bacterial diversity, but also a significant indirect effect on bacterial diversity through influence on microbial antagonistic interactions. Our study reveals the difference among plant species in their effects on soil microbial antagonistic interactions and highlights the vital role of microbial interactions on shaping soil microbial community diversity.

## Introduction

The linkages between above-ground and below-ground communities have been widely studied, and much attention has been paid to the effects of plants on soil microbial communities (Garbeva et al., 2004; Badri and Vivanco, 2009; Bakker et al., 2013a; Schlatter et al., 2015a). Plants strongly influence microbial communities both directly and indirectly. For example, plants provide a wide range of compounds, such as allelopathic constituents (e.g., catechin) and secondary metabolites through root exudation to affect soil microbial communities (Pennanen et al., 1999; Fierer et al., 2012). Moreover, plants may induce competition for nutrients, such as carbon compounds through affecting the amount and quality of litter to influence soil microbial communities (Rousk et al., 2010). Microbe-microbe interactions, such as antagonism and competition, play a significant role in microbial community structure and function (Schlatter et al., 2015b). However, a clearer model is required to allow us to better understand the important role of microbial interactions in regulating the effects of plant species on soil microbial structure.

There are many evidences that plants can significantly influence microbial interactions, which are revealed for instance through assessment of antagonistic activity (Bergsma-Vlami et al., 2005; Bakker et al., 2013b). The plant was suggested to provide environmental context for microbial interactions (Schlatter et al., 2015b). Specifically, plant species significantly impact the availability of soil resources, which may have a direct bearing on resource competition and microbial interactions among soil bacteria (Schlatter et al., 2015b). For instance, plant productivity is suggested to influence microbial competition phenotypes (Wiggins and Kinkel, 2005a). Furthermore, the quality of resources is suggested to mediate microbial interactions (Kinkel et al., 2011, 2012). For example, a higher concentration of phenolic compounds in plant humus reduces microbial activities (Wardle et al., 1998; Inderjit and van der Putten, 2010).

Microbial interactions are broadly perceived to affect the composition and diversity of soil bacterial community (Kinkel et al., 2011; Schlatter et al., 2015b), and are essential for the development and maintenance of microbial communities (Ryan and Dow, 2008). Specifically, soil microbial interactions are expected to change the density and dynamics of microbial populations. To be specific, competing microbial groups can coexist steadily under a certain nutrient concentration ratio, but in the case of nutritional restrictions, certain microbial groups may be defeated (Hibbing et al., 2010). Interspecies signaling molecules, such as sub-inhibitory concentrations of antibiotics, are predicted to mediate the steady state of microbial communities (Davies et al., 2006; Seshasayee et al., 2006). Furthermore, competitive bacteria have been shown to increase bacterial diversity (Domin et al., 2018). Similarly, it has been found that the proportion of soil microbes which have antagonistic activity against other microbes is significantly positively correlated with soil bacterial diversity (Bakker et al., 2013b).

Antagonism is a common phenomenon within soil bacterial communities (Kinkel et al., 2012; Schlatter and Kinkel, 2014; Essarioui et al., 2017), in which one organism causes inhibition of the development or growth of other microorganisms. Such antagonism plays an important role in the formation and maintenance of microbial communities, such as suppressing diverse plant diseases (Chou et al., 2009). *Streptomyces*, a genus of gram-positive bacteria, is ubiquitous in nature (Doumbou et al., 2001) and is well known owing to various metabolic abilities (Bakker et al., 2013b) and producing diverse antibiotics (Kinkel et al., 2012). *Streptomyces* possess diverse antagonistic activities against a variety of plant pathogens among soil bacteria (Jauri and Kinkel, 2014; Takano et al., 2016). Because of their ubiquitous presence and ability to produce antagonistic compounds (Kinkel et al., 2012; Bakker et al., 2013a), *Streptomyces* spp. have been considered as a target for evaluating antagonists (Schlatter et al., 2015b). Studies have shown that microbial antagonism driven by *Streptomyces* is affected by plant species and plant productivity (Wiggins and Kinkel, 2005a; Kinkel et al., 2012; Bakker et al., 2013a). More plant productivity is hypothesized to favor antagonism among soil microbes (Sun et al., 2015).

Although plant species and microbial interactions both have significant impacts on bacterial communities, few studies have explored the role of microbial interactions in regulating the effect of plant species on soil bacterial diversity. In this study, we compared the effects of three dominant shrub species (*Rhododendron simsii*, *Dasiphora fruticosa*, and *Salix oritrepha*) on soil bacterial diversity and microbial interactions in an alpine meadow of north-west China. Shrubs have been shown to positively influence the activity of soil microbial communities (Xu and Coventry, 2003). It has been shown that shrubs support spatial heterogeneity (Grime, 1984), and provide opportunities for a wide variety of interactions, including microbial and plant-microbe interactions (Lopez-Pintor et al., 2006). *Rhododendron simsii*, a kind of evergreen shrub (Kudo et al., 2001; Myers-Smith et al., 2011), produces recalcitrant and uneasily decomposed plant litter (Cornelissen, 1996; Cornelissen et al., 1999), resulting in lower ecosystem productivity (Pastor et al., 1993). Meanwhile, the higher concentrations of phenolic allelochemicals have been found in Rhododendron plants (Wardle et al., 1998) than under other ground-cover taxa (Ward et al., 2009; Adamczyk et al., 2016). *Dasiphora fruticosa* and *Salix oritrepha* are deciduous shrubs (Kudo et al., 2001; Myers-Smith et al., 2011), producing higher quality and more easily decomposed plant litter (DeMarco et al., 2014). Research has shown that dominant plant can modify the diversity of soil microbial communities by impacting soil physicochemical properties through litter inputs (Zhu et al., 2022). The aims of this study were to (1) analyze the bacterial community diversity, microbial antagonistic interaction, and soil properties under different shrub types (i.e., evergreen shrub and deciduous shrubs); and (2) analyze the pathway of deciduous shrubs’ effects on bacterial community diversity compared to evergreen shrub.

## Materials and methods

### Study site

The experiment was conducted at the Gansu Qilianshan National Reserve in Tianzhu (103^°^11′E, 37^°^13′N), Gansu Province, China. The site is located on the northeastern edge of the Tibetan plateau with an average elevation at 2,892 m above sea level. The climate belongs to the alpine arid and semi-arid desert climate. The mean annual temperature ranges from −0.6^°^C to 2^°^C, with approximately 240–270 frost days per year, and the annual precipitation ranges from 300 to 500 mm, falling mainly during the cool summer. The experimental site vegetation is mainly dominated by the shrubs *R. simsii*, *D. fruticosa*, and *S. oritrepha*.

### Experimental design

In early August 2018, we chose a representative area with more than 100 individuals of each of the shrubs *R. simsii*, *D. fruticosa*, and *S. oritrepha*. Then, we randomly allocated 30 plots (30 cm ^∗^ 30 cm) that each included one of the target shrub species. Distance between neighboring plots was less than 5 m, to ensure that the same climatic conditions were present across plots. Thus, a total of 10 individuals were selected for each of the three species of shrub.

Around each selected individual host plant, we randomly collected three soil cores, which were well mixed to form a composite sample. Then all the composite soil samples were divided into three equal-sized aliquots. One aliquot was stored at 4°C and used for evaluating the soil microbial antagonistic potential. One aliquot was stored at −80^°^C for DNA extraction, and the remaining aliquot was used to measure the soil edaphic properties.

### Measuring soil properties

To measure soil water content, 10 g fresh soil was left at 105^°^C for 72 h and the dry weight was determined. The remaining air-dried soil samples were sieved through a 0.15 mm mesh and analyzed for total phosphorus, nitrogen and organic carbon, ammonium, nitrate, and pH. Soil total phosphorus and nitrogen were measured following the semi micro-Kjeldahl protocol and wet oxidation method (Wang et al., 2018). Soil organic carbon was determined based on the wet oxidation method. Both ammonium and nitrate were determined after extracting with 2 M KCl. Soil pH was determined by using a pH meter (PHSJ-3F, Shanghai INESA Scientific Instrument Co., Ltd., China) in a slurry of 1:2.5 (w/v) soil: deionized water (Wang et al., 2018, 2019).

### Soil DNA extraction, qPCR, and sequencing

DNA was extracted using the DNeasy^®^ PowerSoil^®^ Kit (Qiagen, Hilden, Germany) from 0.25 g of each soil samples following the manufacturer’s protocol. For bacterial community composition, the polymerase chain reaction (PCR) was used to amplify the 16S rRNA gene within the V4 hypervariable region with the primers: 515F (GTGCCAGCMGCCGCGGTAA) and 806R (GGACTACHVGGGTWTCTAAT) (Caporaso et al., 2011). The PCR products were visualized using a 2% agarose gel and purified using the GeneJET Gel Extraction Kit (Thermo Scientific), according to the manufacturer’s protocol. The purified 16S rRNA amplicons were sequenced with a lon S5™ XL instrument (Thermo Fisher Scientific). Sequencing data were quality-filtered using Cutadapt v1.9.1 (Jiao et al., 2016), and vsearchGITHUB (Qin et al., 2012) was used to conduct chimera detection. Finally, the high-quality sequences were binned and classified into different operational taxonomic units (OTUs; 97% similarity) by Uparse v7.01 (Rognes et al., 2016). After this process, we obtained 31,212 bacterial OTUs, and bacterial diversity was calculated at the OTU level.

### Microbial antagonism potential

Soil samples were stored at 4^°^C and were processed in random order over 6 months. Each soil sample was evaluated for total *Streptomyces* density, antagonistic *Streptomyces* density, the intensity of inhibition, and frequency of inhibition as described in Bakker et al. (2013b). Due to the ubiquitous presence of *Streptomyces* and the ability to produce antagonistic compounds (Davelos et al., 2004a), *Streptomyces* has been used as a target for evaluating microbial antagonistic interactions (Schlatter et al., 2015b). Briefly, 5 g fresh soil from each sample were dried overnight under sterile cheesecloth, and dispersed in 50 ml H_2_O on a shaker (175 rpm, 60 min, 4^°^C). Soil dilutions were spread on 15 ml agar plates and overlaid with 5 ml of cooled starch-casein agar (SCA) (Wiggins and Kinkel, 2005b). The plates were incubated for 3 days at 28^°^C. After counting the total *Streptomyces* densities, each plate was overlaid with a second thin layer (10 ml) of SCA. Then plates were spread with spore suspensions of each of three indicator *Streptomyces* strains (*Streptomyces olivochromogenes*, *Streptomyces mirabilis*, and *Streptomyces colombiensis*) having different antibiotic resistance profiles, similar to the approach of Davelos et al. (2004b), and were incubated for an additional 3 days at 28^°^C. Zones of inhibition were measured for each plate, and antagonistic *Streptomyces* densities were counted of each plate. Proportions of antagonistic *Streptomyces* were averaged across indicator strains for each sample (Bakker et al., 2013b).

### Data analysis

The Shapiro-Wilk test and Levene’s Test were used to check the assumptions of normal distribution and variance homogeneity among treatments. All indices used in this article met the assumption of normality and equal variance, except the total *Streptomyces* density, antagonistic *Streptomyces* density, soil pH, soil total nitrogen, and soil ammonia nitrogen; for these variables, we assessed impacts of shrub species *via* one-way permutation test. One-way analysis of variance (ANOVA) was used to test the effects of shrub species on the remaining indices, including bacterial Shannon diversity, size of inhibition zone, proportions of antagonistic *Streptomyces*, soil water content, organic carbon, total phosphorus, and nitrate nitrogen. Non-metric multidimensional scaling (NMDS) was used to analyze the structural variation of bacterial communities across samples.

We established theoretical structural equation modeling (SEM) to explore, compared to the evergreen shrub (*R. simsii*), how bacterial diversity and bacterial community composition were impacted by the direct effects of deciduous shrubs (*D. fruticosa* and *S. oritrepha*), and by indirect effects of deciduous shrubs (*D. fruticosa* and *S. oritrepha*) *via* changes in soil microbial antagonism interactions and soil physical-chemical properties (Supplementary Figure 3). The evergreen shrub (*R. simsii*) was used as a control group to explore the ways in which deciduous shrubs (*D. fruticosa* and *S. oritrepha*) affect soil bacterial diversity and bacterial community composition. We used the method as described by Veen et al. (2010) and used PC1 scores (i.e., the first axis of principal components analysis) of total *Streptomyces* density, antagonist density, the intensity of inhibition, and frequency of inhibition to represent microbial antagonistic interactions. Variables were chosen with lower *P*-value according to ANOVA results.

We applied SEM analyses according to the following premises: we hypothesized that (1) There was a significant difference of plant species on microbial antagonistic interaction of associated with *Streptomyces* communities (Bakker et al., 2013b); (2) Plant species will trigger changes in soil physicochemical properties (Bakker et al., 2013a; Wang et al., 2018); (3) Microbial antagonistic interactions are related to the soil microbial diversity (Schlatter et al., 2015b); (4) Soil bacterial diversity is related to the soil physicochemical properties (Herold et al., 2014); and (5) Soil bacterial diversity can be shaped by plant species (Bakker et al., 2013a).

There are many indicators to examine the goodness of fit of SEM, yet there is no single index that is generally accepted. Hence, we used both the χ^2^-test (*P* > 0.05) and the root mean square error of approximation (RMSEA) test to assess the goodness of fit of SEM (Wang et al., 2019). A smaller RMSEA and χ^2^ represents a better model fit. When the RMSEA > 0.06, models should be rejected. If two models have similarly RMSEA and χ^2^, the more parsimonious model is accepted (Danner et al., 2015).

All data analyses were performed in R software, version 4.0.1. The variance homogeneity was tested with the “car” package (Fox et al., 2013). SEMs were conducted using the “lavaan” package (Rosseel, 2012).

## Results

### Shrub species effects on bacterial diversity and bacterial community composition

**FIGURE 1:**
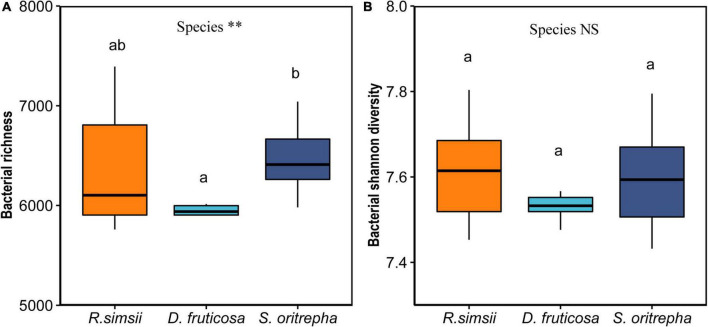
Overview of effects of shrub species on soil bacterial Shannon diversity (mean ± standard error). **(A)** Bacterial richness. **(B)** Bacterial Shannon diversity. Symbol: *^NS^P* > 0.05; ^∗∗^*P* < 0.001 (one-way ANOVA). The same letter means no significant difference (*P* > 0.1).

Targeting the v4 region of the 16S rRNA gene, we obtained 2,054,980 quality-screened sequences from the 30 soil samples using lon S5™ XL sequencing, ranging from 53,142 to 76,845 reads per sample. At the 97% sequence similarity level, 31,212 bacterial OTUs were obtained in total. Shannon index was used to estimate bacterial richness and evenness. Especially, the average values of Shannon index were 7.62 ± 0.04, 7.52 ± 0.03, and 7.60 ± 0.04 (mean ± SE) for soil bacterial communities associated with *R. simsii*, *D. fruticosa*, and *S. oritrepha*, respectively. The richness of soil bacterial community differed significantly among shrub species (Figure 1A). Soil bacterial Shannon diversity did not vary significantly among shrub species (Figure 1B). However, shrub species had significant effects on the composition of bacterial community (Supplementary Figure 1).

### Shrub species effects on soil edaphic properties

**FIGURE 2:**
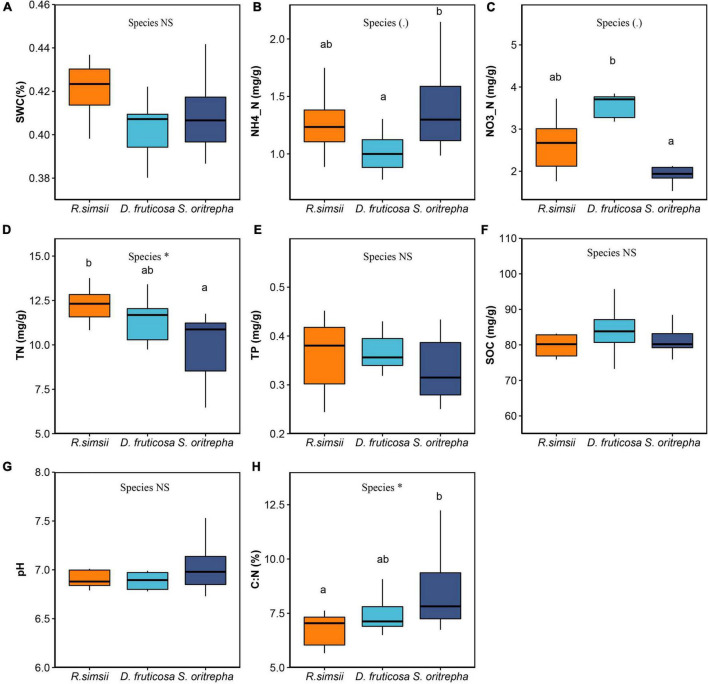
Overview of effects of shrub species on soil edaphic properties. **(A)** Soil water content, **(B)** ammonium, **(C)** nitrate, **(D)** total nitrogen, **(E)** total phosphorus, **(F)** soil organic carbon, **(G)** soil pH, and **(H)** soil carbon-nitrogen ratio. Symbol: ^∗^*P* < 0.05; *^NS^P* > 0.1 (one-way ANOVA). The same letter means no significant difference (*P* > 0.1).

Several measures of soil edaphic properties were significantly impacted by the shrub species (Figure 2). Soil ammonium differed marginally significantly among shrub species (Figure 2B). *S. oritrepha* supported higher soil ammonium than *R. simsii*, and *D. fruticosa* supported lower soil ammonium than *R. simsii*. The soil nitrate also differed among shrub species (Figure 2C), with *S. oritrepha* harboring lower soil nitrate than *R. simsii*, and *D. fruticosa* harboring higher soil nitrate than *R. simsii*. The soil total nitrogen differed among shrub species (Figure 2D), with *D. fruticosa* and *S. oritrepha* supporting lower total nitrogen than *R. simsii*. The soil carbon nitrogen ratio differed significantly among shrub species (Figure 2H), with *S. oritrepha* supporting higher carbon nitrogen ratio than *R. simsii*.

### Shrub species effects on *Streptomyces’*s antagonism potential

Soil total *Streptomyces* density, soil antagonistic *Streptomyces* density, and *Streptomyces* antagonism intensity varied among shrub species. Across all experimental treatments, soil cultivable total *Streptomyces* densities varied nearly twofold, ranging from 2.3 × 10^6^ to 5.7 × 10^6^ colony forming units (CFU) per gram of soil (mean 4.1 × 10^6^ CFU/g). Antagonistic *Streptomyces* densities ranged from 0.9 × 10^5^ to 2.4 × 10^5^ CFU per gram of soil, with a mean of 1.6 × 10^5^ CFU/g. The intensity of inhibition, measured as the average diameter of inhibition zones against indicator strains, varied from 0.47 to 0.78 cm, with a mean value of 0.64 cm. The frequency of inhibition did not change much, from 0.038 to 0.044.

**FIGURE 3:**
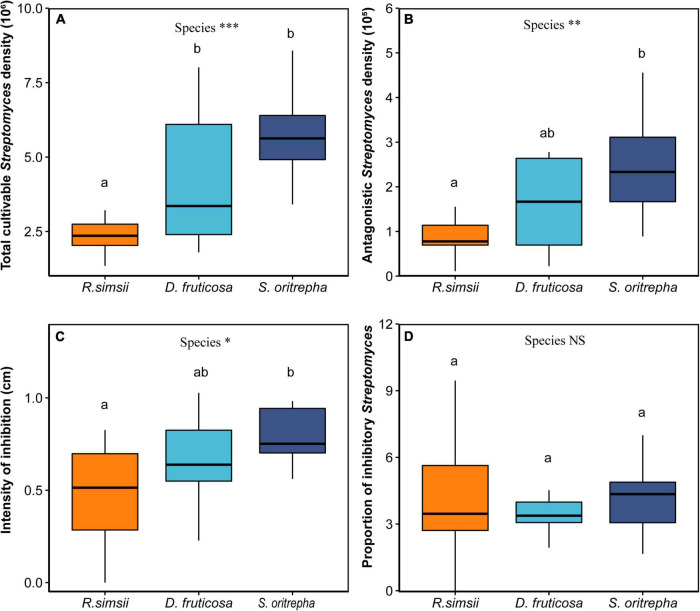
Overview of the effects of shrub species on soil *Streptomyces* antagonism potential (mean ± SE), Effects of plant species on **(A)** the soil total cultivable Streptomyces density, **(B)** the soil cultivable antagonistic *Streptomyces density*, **(C)** the diameter of the inhibition zone, **(D)** the proportion of inhibitory *Streptomyces*. Symbol: ***P < 0.001; **P < 0.01; *P < 0.05; *^NS^P* > 0.1 (one-way ANOVA). The same letter means no significant difference (*P* > 0.1).

Three out of four indices of microbial antagonistic interaction were significantly influenced by shrub species. Specifically, there was a significant difference in soil cultivable total *Streptomyces* density associated with shrub species, the total *Streptomyces* density associated with *R. simsii* was significantly lower than those associated with *S. oritrepha* (Figure 3A, *P* < 0.001, ANOVAP with Kruskal-Wallis Test). Moreover, the soil cultivable antagonistic *Streptomyces* density also differed significantly among shrub species, and antagonistic *Streptomyces* density associated with *R. simsii* was significantly lower than those associated with *S. oritrepha* (Figure 3B, *P* < 0.01, ANOVA with Tukey HSD test). Similarly, there was a significant difference in the diameter of the inhibition zone (Figure 3C, *P* < 0.05, ANOVAP with Kruskal-Wallis Test), and intensity of inhibition associated with these three species of *R. simsii*, *D. fruticosa*, and *S. oritrepha* increased in turn. Generally, the soil total *Streptomyces* density, soil antagonistic *Streptomyces* density as well as the intensity of inhibition consistently showed the same trend of variation among different shrub species, which is the lowest associated with *R. simsii* and the highest associated with *S. oritrepha*. However, there was no significant difference in the proportion of inhibitory *Streptomyces* among shrub species (Figure 3D).

### Direct and indirect shrub species effects on bacterial Shannon diversity

**FIGURE 4:**
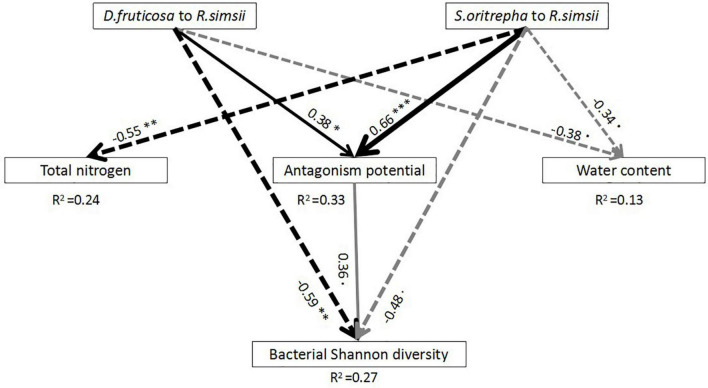
Results of the SEM analyses indicating direct and indirect effects of shrub species on soil bacterial diversity [*P* (Chi-square) = 0.84, df = 3, RMSEA = 0]. Square boxes displayed variables included in the model: effect of *D. fruticosa* compared to *R. simsii*, effect of *S. oritrepha* compared to *R. simsii*, total nitrogen, water content, microbial antagonism potential (PC1 scores of total *Streptomyces* density, antagonistic *Streptomyces* density, intensity of inhibition, and frequency of inhibition), and bacterial diversity. Black arrows indicated significant effects (at the level *P* < 0.05), and gray arrows indicated marginally significant effects (at the level *P* < 0.1). Solid arrows indicated positive effects, while dashed arrows indicated negative effects. *R*^2^ values associated with response variables indicated the proportion of explained variation by relationships with other variables. Values associated with solid arrows represented standardized path coefficients. Symbol: ^∗∗∗^*P* < 0.001; ^∗∗^*P* < 0.01; ^∗^*P* < 0.05.

Our SEM model explained 27% of the variation of bacterial diversity among samples (Figure 4). The SEM showed that compared with *R. simsii*, both *D. fruticosa* and *S. oritrepha* had not only significantly negative direct correlations with bacterial diversity, but also positive indirect correlations with bacterial diversity through *Streptomyces’*s antagonism potential. Specifically, there were significant relationships between *D. fruticosa* and *S. oritrepha* compared to *R. simsii* and *Streptomyces’*s antagonistic interactions, and *Streptomyces’*s antagonism potential were marginally significantly positively related to bacterial diversity. Although *D. fruticosa* and *S. oritrepha* compared to *R. simsii* were significantly negatively related to soil water content, and *S. oritrepha* compared to *R. simsii* was significantly negatively related to soil total nitrogen, there were no significant relationships of soil water content or soil total nitrogen directly on bacterial diversity.

## Discussion

Numerous research results have shown that plant species have significant impacts on the composition and diversity of bacterial communities (Marschner et al., 2004; Costa et al., 2006; De Deyn et al., 2011). Our results showed that there were significant differences in soil bacterial community composition among shrub species (Supplementary Figure 1), and it may be that soil physicochemical properties regulated the effects of shrub species on bacterial community composition. Specifically, compared to *R. simsii*, *S. oritrepha* affected the composition of associated bacterial communities through changing soil total nitrogen (Supplementary Figure 2). However, we did not find that plant species had a significant effect on the soil bacterial Shannon community in our studies (Figure 1). The mechanisms that plant impact soil microbial communities are complex (Bever et al., 2012). Sometimes, although the total effect of plant species on microbial diversity is not significant, the direct or indirect effect are significant (Schlatter et al., 2015a; Wang et al., 2018). Similarly, our result showed that the indirect effects of plants species mediated by *Streptomyces’*s antagonism potential may counteract the direct effects on bacterial diversity (Figure 4).

### Direct effects of shrub species on bacterial diversity

Compared to the evergreen shrub species (*R. simsii*), deciduous shrubs (*D. fruticosa* and *S. oritrepha*) had directly negative effects on bacterial diversity (Figure 4). Many studies showed that the impacts of plant species on soil bacterial diversity depend on the resources provided by the plant species (Sanaei et al., 2022). Kinkel et al. (2011) predicted that high nutrient input would decrease the soil microbial community diversity by decreasing the competition between the microbes in a high density initial soil microbial community. The leaf litter produced by deciduous shrubs is generally easier to decompose than that of evergreen shrubs (DeMarco et al., 2014; Vankoughnett and Grogan, 2016), while evergreen shrubs produce more recalcitrant litter (Cornelissen, 1996; Cornelissen et al., 1999). Further, more easily decomposed plant litter produced by deciduous shrubs is thought to increase soil nutrient storage (Cornelissen et al., 2007). Some studies showed that deciduous shrubs maintain higher rates of primary production compared to evergreen shrubs (van Wijk et al., 2004). Our results showed that the soil organic carbon under deciduous shrubs was higher than under evergreen shrubs, although the difference was not significant (Figure 4). Thus, we inferred that deciduous shrubs (*D. fruticosa* and *S. oritrepha*) induce a higher-resource environment, which directly reduces soil bacterial diversity, compared to the evergreen shrub (*R. simsii*).

### Indirect effects of shrub species on bacterial diversity

Shrub species had indirect effects on bacterial diversity through influence on the antagonism potential of *Streptomyces* (Figure 4). Compared to the evergreen shrub species (*R. simsii*), deciduous shrubs (*D. fruticosa* and *S. oritrepha*) significantly increased the soil microbial antagonism potential driven by *Streptomyces*. It has been demonstrated that resources are an important factor affecting microbial interactions (Kinkel et al., 2012). The majority of the resources obtained by saprotrophic *Streptomyces* are ultimately from plants (Wiggins and Kinkel, 2005a). More importantly, plant root exudation and litter are suggested to modify the effect of plants on microbial interaction (Bakker et al., 2013b). Thus, we inferred that plant characteristics are very likely to affect the potential of microbial antagonism driven by *Streptomyces* (Wiggins and Kinkel, 2005a).

High phenolic-allelopathy (Moral and Cates, 1971) and other secondary compounds (Ward et al., 2009) have been found in evergreen shrubs. It is reported that Rhododendron plants possessed various constituents, and presented significant allelopathic potential (Chou et al., 2009). Rhododendron plants release allelopathic constituents (e.g., catechin) to affect surrounding plants and microorganisms through root exudation and litter (An et al., 2003). Allelopathic constituents are concentrated in plant senescent leaves (Wang et al., 2013), and plant litter decomposition is accompanied by the release of nutrients and allelochemicals. Researchers have proposed that catechin acts as the major allelopathic compound in leaves of Rhododendron plants (Wang et al., 2013); it seems to transform rapidly after being injected into the soil, and subsequently reduces the total cultivable numbers of bacterial community and inhibits the growth of some soil bacterial populations (Inderjit and van der Putten, 2010; Wang et al., 2013). There are also some studies showing that catechin can suppress the growth and activity of microorganisms groups that are sensitive to it (Wang et al., 2013). Thus, we inferred that the allelopathy of the evergreen shrub R. simsii may be the key factor to inhibit the growth of the *Streptomyces* population, as well as inhibiting microbial antagonistic interactions driven by *Streptomyces*. However, more controlled experiments are needed to explore the mechanistic effect of the allelopathy of *R. simsii* on the soil antagonism potential of *Streptomyces*. In addition, compared to the evergreen shrub (*R. simsii*), deciduous shrubs (*D. fruticosa* and *S. oritrepha*) contain lower concentrations of allelopathic constituents (e.g., tannins) and other secondary compounds (Cornelissen et al., 1999; Ward et al., 2009). Therefore, the results showed that *D. fruticosa* and *S. oritrepha* promote the amicrobial antagonistic interaction driven by *Streptomyces*.

Moreover, we found that the diversity of the soil bacterial community was mediated by the antagonism potential of *Streptomyces*. Microbial antagonism has been shown to significantly increase soil bacterial diversity. Several studies have found a close relationship existed in microbial interactions and the composition and diversity of soil bacterial communities (Czaran et al., 2002; Ryan and Dow, 2008; Hibbing et al., 2010; Becker et al., 2012). Research showed that specific genotypes of *Escherichia coli* produce antibiotic colicins, and its toxic effect promotes antagonistic genotype diversity. Likewise, based on a spatially theoretical model, antibiotic interactions within microbial communities have been shown to play an important role in maintaining microbial diversity (Czaran et al., 2002). Additionally, ubiquitous antagonism among the bacterial community (Validov et al., 2005) is hypothesized to favor higher community diversity (Kerr et al., 2002). At the same time, research has shown that a higher proportion of antagonism can significantly increase the diversity of soil bacterial communities (Bakker et al., 2013a). Similarly, a higher frequency of antagonistic bacteria is hypothesized to support the more diverse bacterial communities (Czaran et al., 2002). Therefore, our results are consistent with these findings, and more antagonism activities are expected to increase bacterial diversity.

## Conclusion

Our research provides a better understanding of how plant species directly and indirectly affect bacterial diversity and emphasizes the importance of *Streptomyces’*s antagonism potential in regulating the effects of plant species on soil bacterial diversity. Overall, compared with an evergreen shrub (*R. simsii*), deciduous shrubs (*D. fruticosa* and *S. oritrepha*) decreased bacterial community diversity directly, but they increased bacterial community diversity indirectly through enhancing *Streptomyces’*s antagonistic interactions. Thus, *Streptomyces’*s antagonism potential can offset the directly negative effects of plant species on microbial diversity. This study provides a theoretical basis for studying the microbial interactions that regulate the effects of plant species on soil microbial community diversity.

## Data availability statement

The data presented in this study can be found at the link below: https://datadryad.org/stash/share/tSSena_Xd20RBrgJktKKA7uR0yYxJlxSeIxBinxPGSw.

## Author contributions

SC: conception and design of study. YW, LM, ZL, JC, HS, JW, HC, ZY, SX, and KL: acquisition of data. YW, LM, and SX: analysis and interpretation of data. YW, LM, and SC: drafting the manuscript. All authors contributed to the article and approved the submitted version.
