# Supplementary material for: Microbial interactions play an important role in regulating the effects of plant species on soil bacterial diversity
Source: Front Microbiol. 2022 Sep 15;13:984200. doi: 10.3389/fmicb.2022.984200 (PMC9521175; doi:10.3389/fmicb.2022.984200)
Supplement: Supplementary file 1 [file Table_1.DOCX]

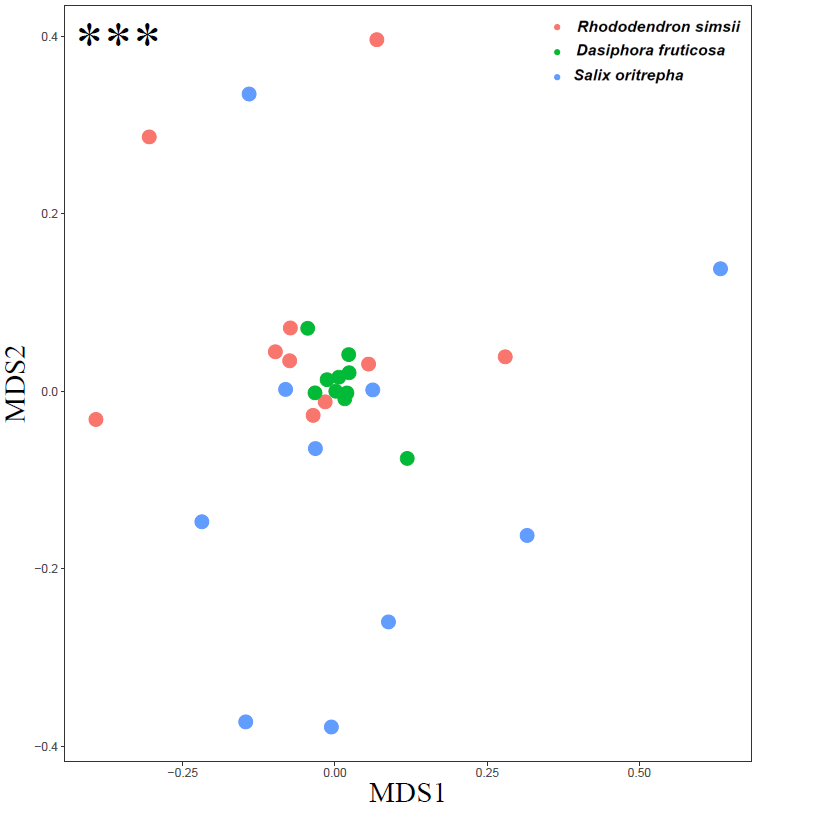


Supplement Figure 1. Nonmetric multidimensional scaling (NMDS) plot derived from bacterial community structure in soil under different plant species. Symbol: ***, P <0.001 (NPMANOVA).


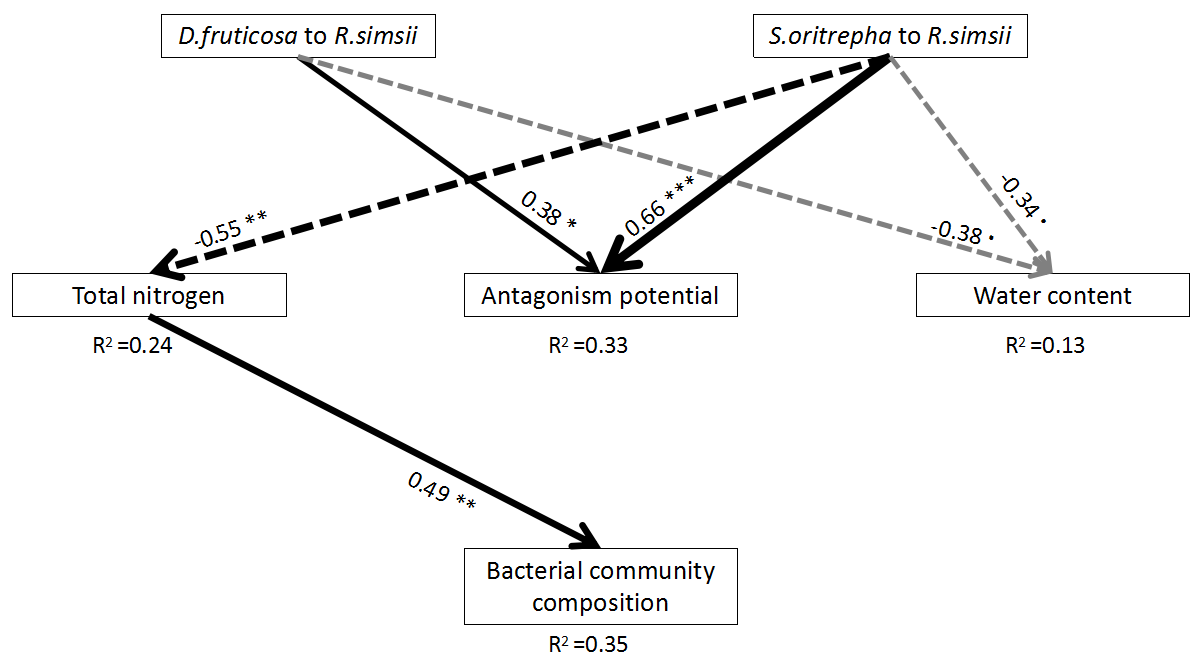


Supplement Figure 2. Results of the SEM analyses indicating indirect effects of shrub species on the composition of bacterial community (*P* (Chi-square) = 0.83, df = 3, RMSEA = 0).


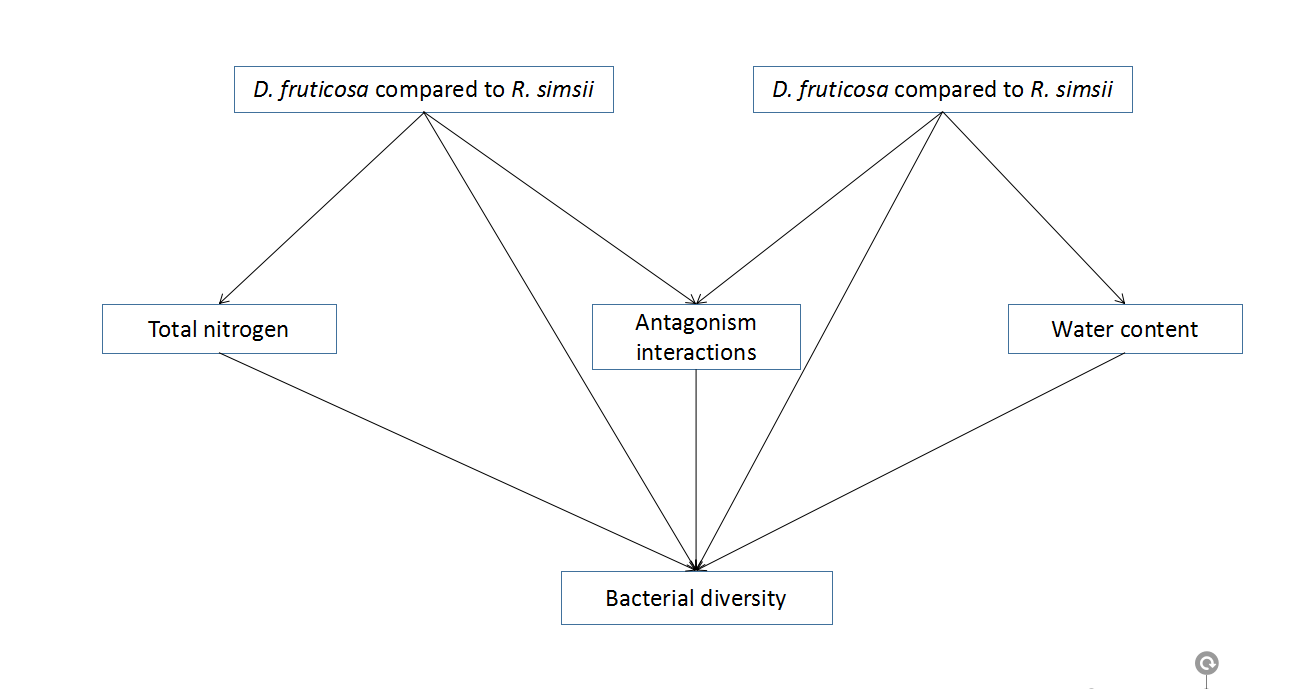


Supplement Figure 3. A priori model of structural equation modelling showing direct and indirect effects of *D. fruticosa* and *S. oritrepha* on soil bacterial diversity, compared to *R. simsii.*
